# A Comparative Study of Risk Factors for Falls in Total Hip and Knee Arthroplasty Patients and Community‐Dwelling Older Adults

**DOI:** 10.1002/msc.70055

**Published:** 2025-01-24

**Authors:** Serena Kuangyi Chen, Don Voaklander, Gian S. Jhangri, C. Allyson Jones

**Affiliations:** ^1^ School of Public Health University of Alberta Edmonton Canada; ^2^ Department Physical Therapy University of Alberta Edmonton Canada

**Keywords:** accidental falls, falls, fear of falling, older adults, total joint arthroplasty

## Abstract

**Objectives:**

Falls in older adults are a public health concern, yet little is known about falls in adults with hip or knee total joint arthroplasty (TJA) who may be at a higher risk than the general population. The study objectives were to compare the number of fallers and fear of falling in TJA patients to age and sex matched community controls, and determine whether the type of risk factors for falls reported in TJA differed from the community group.

**Methods:**

A cross sectional comparative study was conducted with patients waiting or recovering from TJA and age and sex matched comparison group of older adults residing in the community. Reported falls and risk factors for falling were compared to age and sex matched controls from the community. Fear of falling was measured using the Activities‐specific Balance Confidence (ABC) Scale. Logistic regression was used to determine risk factors associated with falls in TJA and community participants.

**Results:**

Of the 198 TJA participants, 29% (*n* = 57) reported falls within the past 12 months compared to 24% (*n* = 24) of 100 participants in the control group (*p* = 0.36). Of those who fell, 25 (44%) were recurrent fallers in the TJA cohort compared with 6 (25%) in the community cohort. Eleven participants reported falls after TJA surgery. Fear of falling was greater in the TJA group (ABC score, mean ± SD: 67.1 ± 24.4) than in the community group (88.1 ± 14.9) (*p* < 0.001).

**Conclusion:**

Although the number of participants who reported falls was comparable in both groups, the TJA group had more recurrent falls, different risk factors for falls, and more fear of falling. Fall prevention programs should be embedded in pre‐operative programs for patients undergoing surgery for TJA.

## 1 Introduction

Falls occur in approximately 30% of older adults (> 65 years) and are the leading cause of fatal and nonfatal injuries (Centres for Disease Control and Prevention, 2018). Osteoarthritis (OA) which is seen in 44% of older Canadians is a painful and debilitating chronic condition (Badley et al. 2019). The odds of falling for a person with OA is nearly 2.5 times that of a person without OA (odds ratio [OR]: 2.4; 95% CI: 1.6, 5.4) (Muir et al. 2010). Falls are typically multifactorial and often include modifiable and non‐modifiable factors (Chen et al. 2019; di Laura Frattura et al. 2018). Several intrinsic risk factors for falls commonly seen with OA include muscle weakness (Chan, Jehu and Pang 2018; de Zwart et al. 2015), balance deficits (Chan, Jehu, and Pang 2018; Levinger et al. 2011), and pain (Chan, Jehu, and Pang 2018; Leveille et al. 2009; Sturnieks et al. 2004); yet radiographic OA has not been recognised as an independent fall risk factor (Cai et al. 2022; Foley et al. 2006).

A subset of adults with OA who may be at even a higher risk of falling, are those who have undergone total hip or knee joint arthroplasty (TJA). When conservative management has been exhausted for OA, TJA is an elective surgical option. In spite of TJA achieving substantial pain relief (Jones and Pohar 2012), rates of physical inactivity (Hammett et al. 2018) and falls are still high both before (23%–63%) and after surgery (12%–38%) (di Laura Frattura et al. 2018). Few extrinsic risk factors in this patient population have been identified (Manlapaz et al. 2019). Extrinsic risk factors for falls that may be applicable to OA include the use of assistive walking devices (Stevens et al. 2009), medications including nonsteroidal anti‐inflammatory drugs (NSAIDs) (Hegeman et al. 2009) and opioids (Yoshikawa et al. 2020), and environmental factors (Canada 2014).

Fear of falling is a modifiable risk factor for falls which is not dependent upon whether a person has fallen or not. This unremitting concern of falling during activity is associated with poor health status, functional decline and overall quality of life (Denkinger et al. 2015; Jørstad et al. 2005; Legters 2002). Several definitions of fear of falling exist; however, the concept includes low confidence in the ability to avoid falls (Legters 2002), which is associated with activity restriction, limited physical activity, poor function, psychological factors such as depression and low confidence in balance (Nguyen et al. 2014). Although high variability of fear of falling (3%–85%) is reported both in older adults who have and have not fallen, variation may be attributable to the characteristics of differing populations and methodologies (Scheffer et al. 2008). Pain and physical limitations seen with OA along with surgical trauma are likely associated with fear of falling with TJA, which may, in turn, impede physical activity during surgical recovery (Jellesmark et al. 2012; Voshaar et al. 2006). Earlier work has reported that many patients are not physically active before and after surgery for TJA (Arnold, Walters and Ferrar 2016; Hammett et al. 2018); however, fear of falling may be a factor that impacts physical activity during the recovery of TJA.

Although several established risk factors for falling have been recognised in older adults (Ganz and Latham 2020), established risk factors for falling with TJA has not been fully elucidated. The identification of modifiable risk factors will be key in targeting specific factors for fall prevention strategies for TJA. The objectives were to compare the number of fallers and fear of falling in TJA patients to age and sex matched community controls, and compare the type the type of risk factors for falls reported by participants for both TJA and community groups.

## 2 Methods

### 2.1 Study Design and Participants

A cross sectional comparative study was conducted with patients waiting or recovering from TJA and age and sex matched comparison group of older adults residing in the community. Age and sex matching was done given that older age and females have a high odds ratio of falling (Deandrea et al. 2010). The TJA cohort was assembled from a large central intake clinic for joint arthroplasty which performs approximately 3500 TJA surgeries annually and serves Edmonton, Alberta, Canada. Participants for the control group were recruited from two Influenza Clinics operated by the provincial health services in Edmonton. Within this universal healthcare system, influenza vaccines were offered to all provincial residents without cost.

Eligibility criteria included participants who (1) were 60 years or older and (2) were English‐speaking. Exclusion criteria were (1) persons whose primary mode of mobility was a wheelchair, and (2) persons with chronic conditions that had high prevalence for falling such as Parkinson disease and multiple sclerosis. Participants from the pre‐operative TJA cohort were consecutively recruited from the pre‐operative educational session for surgery held at the regional joint arthroplasty clinic. Pre‐operative participants were within 2 months of their surgery for TJA. Post‐operative participants were consecutively recruited while they waited for their follow‐up visits with the surgeon. These follow‐up visits occurred within 12 months of surgery. Exclusion criteria for older adults who attended the Influenza Clinics were persons who had a previous hip or knee partial or total arthroplasty and/or were waiting for a TJA. Consecutive sampling was completed at all locations during the Influenza Clinics' working hours. Group‐level matching was done in 5‐year age intervals and sex using a 2:1 ratio for cases to control which resulted in two groups that were comparable in age and sex.

Older adults at the TJA and Influenza clinics were asked to complete the survey while they waited for their appointments. A research assistant was available to answer questions, assist with reading or clarifying survey questions. The survey consisted of 50 questions which included demographics (8 questions), falls and circumstances of falls (10 questions), fear of falling (Activities‐specific Balance Confidence [ABC]) scale (Powell and Myers 1995), and 33 established risk factors for falling (Scott et al. 2007) including intrinsic (e.g. medical) and extrinsic factors (e.g., use of walking devices). The majority of questions were close‐ended (multiple‐choice) to standardise responses and improve survey completion (Kitchenham and Pfleeger 2002). Ethics approval was obtained through the University of Alberta Health Research Ethics Board (PRO 00065389).

*Fall Status*: While a variety of definitions exist as to what constitutes a fall (Tinetti, Speechley and Ginter 1988), we chose to use a comprehensive, non‐exclusive falls definition that is simple and understood by lay people (Hauer et al. 2006). A fall was defined as ‘a sudden loss of balance or gait that leads you to land on the ground or a lower level than where you were originally’ (Tinetti, Speechley, and Ginter 1988). Although a recall period of 12 months for falls was used, we also asked participants who were recovering from TJA whether they fell after surgery. Circumstances of the falls such as location, related injuries, and medical attention were also documented.

*Fear of falling*: The Activities‐Specific Balance Scale (ABC) Scale (Powell and Myers 1995) was used to evaluate balance confidence, that is, the level of confidence in a participant's ability to maintain their balance and remain steady while performing various activities. The 16‐item measure generates an overall balance confidence score that ranges from 0 to 100, with higher scores indicative of greater confidence with balance. Although no minimal important difference (MID) exist for ABC Scale, Lajoie and colleagues identified a cut‐off score below 67% as predictive of future falls in older adults (Lajoie and Gallagher 2004). Participants also completed rating fear of falling, defined as a persisting concern regarding falling, using a four point Likert scale asking, ‘are you afraid of falling?’ with choices ranging from: ‘not at all afraid’, ‘slightly afraid’, ‘somewhat afraid’, or ‘very afraid’ (Howland et al. 1993).

### 2.2 Statistical Analysis

Participants were classified into 3 groups: non‐fallers, single fallers and recurrent fallers (2+ falls within the past 12 months). Others have recommended distinguishing falls according to the number of falls since recurrent fallers are more likely to benefit from fall prevention strategies (Fletcher et al. 2009). Summary statistics were completed for all variables. Group comparison tests such as Students *t*‐tests were used to identify group differences. Because the objective was to identify established risk factors for falls (Ganz and Latham 2020), simple logistic regression models were performed for each of the known risk factors. Two‐tailed tests with *p*‐values of 0.05 were considered statistically significant. Data were analysed using Stata 14 Statistical Software (StataCropt 2015).

## 3 Results

The TJA group consisted of 198 participants, of which the mean age was 71.2 ± 6.6 years and 117 (59%) were female. There were 94 (47.5%) participants waiting or received a total hip arthroplasty (THA) while 104 (52.5%) participants comprised the total knee arthroplasty (TKA) subset. The pre‐operative participants consisted of 114 (57.6%; *n* = THA 60; TKA 54) and another 84 (42.4%; *n* = THA 34; TKA 50) were recovering from surgery. The community cohort (*n* = 100) had a mean age of 71.4 ± 5.7 years with 59% (*n* = 59) female. While age and sex of the TJA and community groups were comparable, the TJA group had a higher body mass index (BMI) (30.5 kg/m^2^ ± 5.7; community: 27.4 kg/m^2^ ± 5.2; mean difference: 2.93 kg/m^2^, 95% CI: 1.58–4.28, *p* < 0.01), and more self‐reported chronic conditions (3.1 ± 2.0; community: 2.3 ± 1.8, *p* < 0.01). A higher proportion of the TJA cohort used walking devices than the community group (*p* < 0.01). Only 12% (*n* = 35) of the TJA group ambulated independently without an assistive device, and 163 participants reported use of assistive devices such as a walker (*n* = 68, 34%) or a cane/crutch (*n* = 89, 45%). Twenty‐two percent (*n* = 41) of the TJA group were able to walk more than 6 blocks compared to 81% (*n* = 81) of the participants in the community group (*p* < 0.01). The community group did not use walkers except for three participants and 88 (88%) participants ambulated independently (Table 1).

**TABLE 1:** Characteristics between total joint arthroplasty and community participants.

	Total joint arthroplasty *n* = 198	Community *n* = 100	*p*‐value
Age, years, mean (SD)	71.2 (6.6)	71.4 (5.7)	0.79
Sex, female, *n* (%)	117 (59%)	59 (59%)	0.98
Marital status, *n* (%)			< 0.01
Married/common law	151 (76%)	64 (64%)	
Divorced/widowed	46 (23%)	26 (26%)	
Never married/single	1 (0.5%)	10 (10%)	
Employment, *n* (%)			0.06
Retired/disability leave	164 (83%)	91 (91%)	
Full time/part time/casual	34 (17%)	9 (9%)	
Education years, mean (SD)	13.4 (8.4)	14.0 (3.1)	0.37
Medical			
Body mass index, kg/m^2^, mean (SD)	30.5 (5.7)	27.4 (5.2)	< 0.01
THA, *n* (%)	88 (44%)	N/A	
TKA, *n* (%)	110 (56%)	N/A	
Other joint involvement, n	88	0	
Previous hip replacement, *n* (%)	39 (44%)	N/A	
Previous knee replacement, *n* (%)	49 (56%)	N/A	
Pre‐operative	114	N/A	
Post‐operative	84	N/A	
Comorbid conditions, mean (SD)	3.1 (2.0)	2.4 (1.8)	< 0.01
Medications			
Number of medications, mean (SD)	1.8 (1.4)	1.1 (1.4)	< 0.01
Pain, *n* (%)	114 (57%)	17 (17%)	< 0.01
High blood pressure, *n* (%)	25 (13%)	12 (12%)	0.80
Sedatives or hypnotics, *n* (%)	10 (5%)	2 (2%)	0.20
Anxiety medication, *n* (%)	10 (5%)	1 (1%)	0.08
Ambulatory status			
Walking devices, *n* (%)			< 0.01
None	67 (34%)	88 (88%)	
Uses canes	101 (51%)	9 (9%)	
Uses crutches	13 (10%)	0	
Uses walker	41 (21%)	3 (3%)	
Walk distance, *n* (%)			< 0.01
Unlimited	24 (12%)	66 (66%)	
6–10 blocks	19 (10%)	15 (15%)	
1–5 blocks	66 (33%)	14 (14%)	
Less than 1 block	52 (26%)	3 (3%)	
Indoors only	36 (18%)	2 (2%)	
Limits to walking			< 0.01
Pain, *n* (%)	146 (74%)	21 (21%)	
Fatigue, *n* (%)	45 (22%)	25 (25%)	
No limits, *n* (%)	18 (9%)	54 (54%)	
Flights of stairs able to climb, mean (SD)	1.6 (2.1)	3.7 (3.1)	< 0.01
Fear of falling			
Activities specific balance (ABC) scale, mean (SD)	67.0 (24.3)	88.4 (14.9)	< 0.0001
ABC scale score, *n* (%)			< 0.001
ABC ≥ 67%	108 (55%)	92 (92%)	
ABC < 67%	90 (45%)	8 (8%)	
Self reported fear of falling			< 0.001
Not afraid (not at all afraid/a bit)	121 (61%)	83 (83%)	
Afraid (very afraid/somewhat afraid)	77 (39%)	17 (17%)	

### 3.1 Falls and Circumstances of Falls

Within the TJA group, 57 (29%) participants reported falling over the past year, of which 25 (44%) participants reported more than 1 fall and were considered recurrent fallers (range 2–6 falls). Of the 114 pre‐operative participants, 34 (30%) reported falling in the past 12 months, the majority of falls occurred outdoors (*n* = 16, 67%). Of the 18 falls reported by post‐operative participants, 11 (13%) reported a fall after surgery with five reporting falls outdoors. Within the community group, 24 (24%) reported falling in the past year of which 6 (25%) were considered recurrent fallers (range 2–6) (Table 2).

**TABLE 2:** Falls, fear of falling, and circumstances of falls in total joint arthroplasty participants, and community controls.

	Total joint arthroplasty (*n* = 198)	Community (*n* = 100)
Number of fallers	57 (29%)	24 (24%)
Recurrent fallers (2 or more)	25/57 (44%)	6/24 (25%)
Patients who fell after surgery	11/84 (13%)	—
Total number of falls	96	38
Single fall	28	17
Recurrent falls	68	21
Location of falls, *n* (%)		
Indoors	26 (37%)	5 (19%)
Outdoors	42 (60%)	21 (81%)
Hospital	1 (1%)	0
Fall‐related injuries:		
Sprain/strain/bruises/cuts	28	8
Head injuries	3	1
Fracture	4	1
Medical attention from a health professional within 48 h? (Yes), *n* (%)	18 (18%)	3 (8)%

## 4 Fear of Falling

The mean ABC Scale score for the TJA group was 67.1 ± 24.4, as compared to a higher mean score for the community group (88.4 ± 14.9, *p* < 0.01) which was indicative of greater confidence in balance (Table 2). ABC Scale scores were lower for fallers (mean difference 9.5 ± 3.1, 95% CI: 3.4–15.5, *p* = 0.02) regardless of TJA or community groups (Table 2). Within the TJA group who reported falls (*n* = 57), 33 (57%) had ABC Scale scores below 67% (mean ABC Scale score 44.4 ± 17.1) as compared to 24 (42%) who had not fallen (mean ABC Scale score 82.9 ± 9.8). Recurrent TJA fallers (*n* = 25) were more fearful of falls (mean ABC Scale score 52.76 ± 25.4) as compared to one‐time fallers and non‐fallers (*n* = 173) (mean ABC Scale score 69.12 ± 23.6) (*p* < 0.01). Forty‐five percent (*n* = 90) of the TJA participants scored below 67% on the ABC scale, which is predictive of falls, as compared to 7% (*n* = 7) in the community group. A higher percentage (39%) of the TJA group as compared to 17% of the community group reported being afraid of falling.

### 4.1 Risk Factors Associated With Falls

The most frequently reported risk factors in the TJA group were: taking one to three medications (*n* = 137, 69%), having more than three co‐morbid conditions (*n* = 117, 59%) with arthritis as the most commonly reported chronic condition (*n* = 120, 61%), and use of assistive devices (130, 66%), of which 45% (*n* = 89) reported using a cane or crutch. In the community group, the most commonly reported risk factors were: taking at least one medication (*n* = 63, 63%), and having more than three co‐morbid conditions (*n* = 40, 40%) with high blood pressure as the most frequently reported co‐morbid condition (*n* = 44, 44%).

Using logistic regression, the risk factors in the TJA cohort that had the highest odds of falling were the use of more than four medications (OR 3.8, 95% CI: 1.1–13, *p* = 0.03), the use of a walker (OR 2.8, 95% CI: 1.1–6.7, *p* = 0.03), self‐reported ‘very afraid’ or ‘somewhat afraid’ of falling (OR 2.3; 95% CI: 1.2–4.6, *p* = 0.01), ABC below 67% (OR 2.1; 95% CI 1.1–4.0), and high blood pressure (OR 2.0; 95% CI: 1.1–3.9, *p* = 0.03) were factors associated with falls. Factors associated with fallers in community ‐ dwelling older adults differed from the TJA group in that many risk factors were medically related, including mental health problems (OR 8.0; 95% CI: 1.3–50, *p* = 0.03), and urinary or bowel incontinence (OR 5.1, 95% CI: 1.1–22, *p* = 0.03) (Table 3). Having more than three co‐morbid conditions were associated with falls in both the TJA group (OR 2.2; 95% CI: 1.5–6.5, *p* < 0.01) and the community group (OR 1.4; 95% CI: 1.1–7.5, *p* = 0.03).

**TABLE 3:** Factors associated with falls in the total joint arthroplasty group, and the community group.

	*n* (198)	Total joint arthroplasty (OR (95% CI, *p*)	*n* (100)	Community OR (95% CI, *p*)
Number of comorbid conditions				
0–3	81	1.0 (reference)	60	1.0 (reference)
3+	117	2.2 (1.5–6.5, < 0.01)	40	1.4 (1.1–7.5, 0.03)
Eye problems	41	1.3 (0.6–2.9, 0.47)	24	1.4 (0.5–4.2, 0.46)
Fear of falling				
Not afraid of falling (not at all afraid/a bit)	121	1.0 (reference)	83	1.0 (reference)
Afraid of falling (very afraid/somewhat afraid)	77	2.3 (1.2–4.6, 0.01)	17	1.1 (0.4–4.8, 0.58)
ABC scale score				
ABC ≥ 67%	108	1.0 (reference)	92	1.0 (reference)
ABC < 67%	90	2.1 (1.1–4.0, 0.02)	8	1.7 (0.3–8.7)
Medications				
None	37	1.0 (reference)	45	1.0 (reference)
1–3	137	2.1 (0.8–5.6, 0.19)	63	2.1 (0.8–5.5, 0.10)
4+	24	3.8 (1.1–13, 0.03)	6	3.4 (0.5–24, 0.20)
Ambulation				
Distance able to walk				
More than 6 blocks	43	1.0 (reference)	81	1.0 (reference)
1–5 blocks	66	2.3 (0.9–5.9)	14	3.4 (1.0–11.2)
Unable to walk/indoors/< 1 block	88	2.1 (0.8–5.2)	2	a
Walking devices				
No device	68	1.0 (reference)	89	1.0 (reference)
Cane or crutch	89	1.1 (0.5–2.3)	8	a
Walker	41	2.8 (1.1–6.7, 0.03)	3	a

^a^ Unable to calculate due to too few numbers.

## 5 Discussion

Approximately one‐third of participants who were pre‐ and post‐operative TJA reported falls over the past year. The proportion of TJA participants who fell in this cohort is similar to rates reported in TJA by others, 17%–40% (Chan, Jehu, and Pang 2018; Tsonga et al. 2016; Wu et al. 2018). Although it is reasonable to assume that people with TJA would have more falls than community dwelling older adults given the number of additional risk factors associated with pain, ambulation and presence of co‐morbid conditions, the number of participants who had any fall was comparable between the two groups in this study. A smaller study (*n* = 62) also found that the proportion of reported falls over a 12 month period in people waiting for TKA (17/35, 48%) was similar to an age‐matched community group (8/27, 30%); however, recurrent falls were not identified (Levinger et al. 2011).

In spite of comparable proportion of falls in TJA and community control groups, a higher proportion of recurrent falls were reported with the TJA group than the community cohort. Earlier studies of older adults have highlighted the difference in risk of falls between one‐time fallers and recurrent fallers (Campbell et al. 1981; Masud and Morris 2001) who are regarded at ‘high risk’ (Montero‐Odasso et al. 2022). Others have reported an increased likelihood of recurrent falls was related to the coexistence of sarcopenia and OA (Iijima and Aoyama 2021) which may, in part, explain recurrent falls with advanced OA and TJA. While recurrent falls are regarded as an indicator of poor health, this applies more so for indoor falls than outdoor falls (Kelsey et al. 2012). In our Canadian cohort that must contend with 4 distinct seasons, however, the majority of falls occurred outdoors for both the TJA and community control groups. Regardless of the location of falls, guidelines recommend that people who fall frequently (≥ 2 falls) require a comprehensive multifactorial falls risk assessment to guide a person‐centred intervention (Eckstrom et al. 2024; Montero‐Odasso et al. 2022).

Another modifiable risk factor specific to TJA was the use of walking devices, in particular the use of a walker. The use of walkers is associated with more falls and fall‐related injuries in older adults over 65 years treated in the emergency department for nonfatal fall injuries (rate ratio 2.6) (Stevens et al. 2009). In a review, Bateni and Maki reported that when canes and walkers were used improperly they were associated with increased risk of falling (Bateni and Maki 2005). Proper fitting and instructions on the use and safety of walkers during the preoperative and postoperative periods may be a simple yet effective method to reduce falls in this patient population.

In spite of large improvements in physical function and pain relief after TJA, patients show little improvement in physical activity (Almeida, Khoja and Piva 2018; Hammett et al. 2018), which may be indirectly related to fear of falling (Sawa et al. 2018; Tsonga et al. 2016). According to other studies, up to 57% of independently living older adults aged 60 years or older feel some degree of fear for falling (Friedman et al. 2002; Howland et al. 1998; Yardley and Smith 2002) and up to 88% in older women with knee OA (Fernandes et al. 2024). Within our TJA cohort reported some degree of fear of falling using a four‐point Likert scale, and approximately half of participants had ABC scores that were predictive of falls (Lajoie and Gallagher 2004) which was greater than in the community control group. Higher fall‐related self‐efficacy and confidence in performing certain daily tasks without losing balance has been associated with higher levels of activity function, performance, and fewer falls (Schepens et al. 2012). A fear of falling that leads to limited physical activity can potentially exacerbate comorbidities associated with OA such as decreased mobility (Deandrea et al. 2010), muscle weakness (Moreland et al. 2004), and depression or anxiety (Painter et al. 2012), all of which, in turn, may increase a person's risk of recurrent falls (Prusinowska et al. 2016). Perhaps more importantly, fear of falling is a key feature in management of falls prevention (Tinetti, Speechley, and Ginter 1988). Although no clear recommendation for one clinical intervention targeting fear of falling has been identified, healthcare clinicians are encouraged to assess fear of falling and implement a multicomponent programme that consists of exercise, education and cognitive behavioural therapy for those patients with higher fear of falling (Kaya and Bilik 2022; Whipple, Hamel and Talley 2018).

A primary strength of this study was the use of a comparable community group of older adults, which allowed direct comparison of fall rates in older adults. Few studies that examined TJA and falls included a comparison group (Ikutomo et al. 2015; Mitchell et al. 2007; Swinkels, Newman and Allain 2008). Secondly, TJA participants were recruited from a regional site that performed in excess of 3500 primary TJA annually. This recruitment site yielded more generalisable findings and minimised referral bias compared to studies conducted at a single site or involving a single surgeon. In light of these strengths, some limitations warrant further discussion. Firstly, the cross‐sectional study design did not allow us to identify the causal mechanism of known risk factors associated with falling. Another limitation which is inherent to many studies examining falls was ascertainment of falls. Our recall time may have affected the recall of falls, although a 1‐year recall time is more accurate than a shorter recall time of three or 6 months (Hale, Delaney and Cable 1993). It is also likely that falls were under‐reported given the recall period and the stigma associated with falling (Hoffman et al. 2018). With technological advancements in accelerometers and smart phones, wearable devices that monitor activity may be a more rigorous option for measuring falls in the community (Habib et al. 2014).

Findings from this survey suggest that while the proportion of fallers is comparable between TJA patients and community older adults, recurrent falls were more frequently reported with TJA patients. Fearful of falling was greater in TJA patients who were regarded as high risk than one‐time fallers or non‐fallers. Multimorbidity and specific chronic conditions such as vision, along with the number of medications were significant factors for both the TJA and community groups and are recognised within the World Guidelines for falls prevention and management (Montero‐Odasso et al. 2022). Modifiable risk factors of falls identified in this TJA cohort were fear of falling and walking devices. Given that effective multifactorial interventions exist for fall prevention including fear of falling and the use of walking devices in clinical and community settings (Brouwer et al. 2003; Gillespie et al. 2012; Grossman et al. 2018; Vlaeyen et al. 2015), a person‐centred falls prevention strategy that targets modifiable risk factors in patients undergoing TJA should be a mandatory component of pre‐operative patient education.

## Author Contributions

All authors were involved in drafting the article or revising it critically for important intellectual content, and all authors approved the final version to be published. S.K.C. has full access to all of the data in the study and takes responsibility for the integrity of the data and the accuracy of the data analysis.

## Conflicts of Interest

The authors declare no conflicts of interest.

## Data Availability

Research data are not shared.
